# Quorum quenching of *Streptococcus mutans* via the nano-quercetin-based antimicrobial photodynamic therapy as a potential target for cariogenic biofilm

**DOI:** 10.1186/s12866-022-02544-8

**Published:** 2022-05-10

**Authors:** Maryam Pourhajibagher, Mojgan Alaeddini, Shahroo Etemad-Moghadam, Bahman Rahimi Esboei, Rashin Bahrami, Rezvaneh sadat Miri Mousavi, Abbas Bahador

**Affiliations:** 1 Dental Research Center, Dentistry Research Institute, Tehran University of Medical Sciences, Tehran, Iran; 2 Department of Parasitology and Mycology, School of Medicine, Tonekabon Branch, Islamic Azad University, Tonekabon, Iran; 3 Department of Orthodontic, School of Dentistry, Tehran University of Medical Sciences, Tehran, Iran; 4 Pharmaceutical Engineering Laboratory, School of Chemical Engineering, College of Engineering, University of Tehran, Tehran, Iran; 5 Fellowship in Clinical Laboratory Sciences, BioHealth Lab, Tehran, Iran

**Keywords:** Antimicrobial photodynamic therapy, Competence-stimulating peptide, Dental caries, Quorum sensing, *Streptococcus mutans*

## Abstract

**Background:**

Quorum sensing (QS) system can regulate the expression of virulence factors and biofilm formation in *Streptococcus mutans*. Antimicrobial photodynamic therapy (aPDT) inhibits quorum quenching (QQ), and can be used to prevent microbial biofilm. We thereby aimed to evaluate the anti-biofilm potency and anti-metabolic activity of nano-quercetin (N-QCT)-mediated aPDT against *S. mutans*. Also, in silico evaluation of the inhibitory effect of N-QCT on the competence-stimulating peptide (CSP) of *S. mutans* was performed to elucidate the impact of aPDT on various QS-regulated genes.

**Methods:**

Cytotoxicity and intracellular reactive oxygen species (ROS) generation were assessed following synthesis and confirmation of N-QCT. Subsequently, the minimum biofilm inhibitory concentration (MBIC) of N-QCT against *S. mutans* and anti-biofilm effects of aPDT were assessed using colorimetric assay and plate counting. Molecular modeling and docking analysis were performed to confirm the connection of QCT to CSP. The metabolic activity of *S. mutans* and the expression level of various genes involved in QS were evaluated by flow cytometry and reverse transcription quantitative real-time PCR, respectively.

**Results:**

Successful synthesis of non-toxic N-QCT was confirmed through several characterization tests. The MBIC value of N-QCT against *S. mutans* was 128 μg/mL. Similar to the crystal violet staining, the results log_10_ CFU/mL showed a significant degradation of preformed biofilms in the group treated with aPDT compared to the control group (*P* < 0.05). Following aPDT, metabolic activity of *S. mutans* also decreased by 85.7% (1/2 × MBIC of N-QCT) and 77.3% (1/4 × MBIC of N-QCT), as compared to the control values (*P* < 0.05). In silico analysis showed that the QCT molecule was located in the site formed by polypeptide helices of CSP. The relative expression levels of the virulence genes were significantly decreased in the presence of N-QCT-mediated aPDT (*P* < 0.05).

**Conclusions:**

The combination of N-QCT with blue laser as a QQ-strategy leads to maximum ROS generation, disrupts the microbial biofilm of *S. mutans*, reduces metabolic activity, and downregulates the expression of genes involved in the QS pathway by targeting genes of the QS signaling system of *S. mutans*.

## Introduction

Dental caries is a common irreversible chronic infectious disease that over time, demineralizes the tooth structure and results in progressive tooth decay [1]. The dental cariogenic pathogen *Streptococcus mutans* synthesizes quorum sensing (QS) signals, competence-stimulating peptides (CSP) that are sensed via a two-component signal system (ComDE) [2]. The *comC* gene encodes the ComC propeptide, that is processed by the ABC transporter complex, ComAB, leading to the production of 21-CSP. This 21-amino acid polypeptide is cleaved into an 18-amino acid polypeptide (18-CSP) by SepM, a membrane-localized protease. 18-CSP, activates the cytoplasmic response regulator ComE through interaction with ComD, a histidine kinase membrane-bound protein receptor. Active ComE then results in the expression of various virulence genes as a response to the development of competence [3–5]. A previous study showed that the ComD/ComE signal transduction system can regulate the expression of glucosyltransferase B/C/D (*gtfB/C/D*), fructosyltransferase (*ftf*), and glucan-binding protein B (*gbpB*) genes [6, 7].

*S. mutans* utilizes Com-dependent QS systems to coordinate a myriad of biological processes such as regulating genetic transformation, natural competence, niche adaptation during host colonization, sporulation, virulence, and biofilm formation [5]. The structure of *S. mutans* biofilm acts as a barrier in exposure to the chemical anti-biofilm agents and leads to increase resistance against different antimicrobial factors. It has been reported that any mechanism with the capability of inhibiting any one of the key processes in the QS signals, named quorum quenching (QQ) can be potentially used for QQ sensing and preventing microbial infections. Given that research in QS inhibitors and QQ has been progressing so rapidly in recent years, the introduction of new mechanisms of QQ is inevitable [8, 9].

According to the literature, antimicrobial photodynamic therapy (aPDT) by suppression of the virulence factors of microbial pathogens is a novel approach to inhibit biofilms [10–12]. aPDT is a multi-stage process including administration of a non-toxic dye called photosensitizer (PS), harmless visible light irradiation with specific wavelengths for PS, and interaction of the excited state with oxygen. The excited PS can produce reactive oxygen species (ROS) and result in microbial cell death either by damaging the cell membrane or proteins, lipids, and deoxyribonucleic acid (DNA). Up to now, a variety of in vitro and in vivo aPDT studies have demonstrated a broad-spectrum of activity in favor of biofilm-eradication or substantial reduction [13].

As mentioned, one of the most important components involved in aPDT is PS. Among PS structures commonly employed in aPDT, natural products have received a lot of attention. Quercetin (QCT) as a major flavonoid, a class of secondary metabolic products of plants, possesses a wide range of pharmacological activities, including neuroprotective, antioxidant, anticancer, antimicrobial infections, and antiapoptotic applications [14]. QCT has two maximum absorption bands 380 and 258 nm [15] with a strong biological effect at micromolar concentrations after activation by light at 405 ± 10 nm.

Due to the increasing need for the effective degradation of *S. mutans* biofilm in dental caries, more attention in this study has been focused on the combination of nano-QCT (N-QCT) and blue laser light. Since previous studies have shown that aPDT can be a useful supplementary strategy for QS inhibitor in inducing microbicidal effect and reducing the possibility of drug resistance [16–18], the current study was designed (i) to determine the anti-biofilm potency and anti-metabolic activity of N-QCT-mediated aPDT against *S. mutans*, (ii) to identify in silico N-QCT with an inhibitory effect on *S. mutans* CSP, and (iii) to elucidate the effects of aPDT on various QS regulated genes. It was hypothesized that the combination of phototherapy and N-QCT can enhance the photosensitivity of *S. mutans* biofilm in response to aPDT.

## Materials and methods

### Synthesis of N-QCT

N-QCT was synthesized according to the study by Debnath et al. [19] with a slight modification. Briefly, QCT (Sigma-Aldrich, Germany) solution at a concentration of 3 mg/mL was prepared in 1% DMSO (Merck, Germany). Then, 30 mL of poly(vinyl alcohol) (PVA, Sigma-Aldrich, Germany) 0.1% (w/v) was added and the resulting mixture was magnetically stirred at room temperature for 6 h to form a colloidal suspension. The suspension was subsequently centrifuged at 15,000 rpm for 15 min to remove free un-encapsulated QCT. The final product was transferred to a freeze-dryer to obtain a dry powder.

### Characterization of synthesized N-QCT

The surface morphology of N-QCT was studied by field emission scanning electron microscopy (FESEM; ZEISS, German). The size distribution profiles of nanometer-sized particles and zeta potential of N-QCT were carried out using a MALVERN Zetasizer Ver. 6.01 (Malvern Instruments, UK) at approximately 25 °C. Energy-dispersive X-ray spectroscopy was used to analyze the elemental composition of N-QCT. Also, the entrapment efficiency (EE%) and drug loading (DL%) of N-QCT were determined by an ultraviolet-visible (UV–vis) spectroscopy (Eppendorf BioSpectrometer®, Germany) at 438 nm using the following equations:

$$\mathrm{EE}\%=\frac{\mathrm{Total}\ \mathrm{amount}\ \mathrm{of}\ \mathrm{QCT}-\mathrm{Free}\ \mathrm{QCT}}{\mathrm{Total}\ \mathrm{amount}\ \mathrm{of}\ \mathrm{QCT}}\times 100$$

and

$$\mathrm{DL}\%=\frac{\mathrm{Total}\ \mathrm{amount}\ \mathrm{of}\ \mathrm{QCT}-\mathrm{Free}\ \mathrm{QCT}}{\mathrm{N}-\mathrm{QCT}\ \mathrm{weight}}\times 100$$

### Absorption spectra of N-QCT

The ultraviolet-visible (UV–vis) spectra of N-QCT were scanned within the wavelength range of 300–600 nm using an UV–vis spectrophotometer.

### Cytotoxicity and N-QCT induced cell survival assay

The normal human gingival fibroblast (HGF) cells (IBRC C10459) at a density of 1 × 10^5^ cells/well were sub-cultured in a 96-well culture plate containing 200 μL of Dulbecco’s Modified Eagle Medium (DMEM) supplemented with 10% fetal bovine serum (FBS), 2 mM L-glutamine, 100 μg/mL amphotericin B, and 1% penicillin/streptomycin antibiotic solution (10,000 Unit/mL penicillin, 10 mg/mL streptomycin). The cells were incubated overnight in a humidified incubator at 37 °C with 5% CO_2_ and 95% air for adherence to the culture plate. Afterward, the cells were incubated at 37 °C for 24 h in presence of N-QCT at the different concentrations (128, 256, and 512 μg/mL) and cell viability was determined by MTT assay as reported previously [20].

### Bacterial strain and growth conditions

The standard strain of *S. mutans* ATCC 35668 was cultured from single colonies in brain heart infusion (BHI) broth (Sigma-Aldrich, Germany) at 37 °C in a 5% CO_2_, aerobic atmosphere. Bacterial suspensions were standardized with half McFarland standard, equivalent to a suspension of 1.5 × 10^8^ colony forming unit (CFU) per milliliter (mL).

### Determination of Minimum Biofilm Inhibitory Concentration (MBIC) of N-QCT

The broth microdilution method was used for determining the MBIC dose of N-QCT against *S. mutans* according to Clinical and Laboratory Standards Institute (CLSI) guidelines [21]. We used the experimental conditions of the method described by Aires et al. [22] to prepare *S. mutans* biofilms. Briefly, 100 μL of bacterial cells at a final concentration of 1.5 × 10^6^ CFU/mL was added to wells of a 96-well microtiter plate which were containing two-fold N-QCT-dilutions ranging from 512 to 1.0 μg/mL in ultrafiltered buffered tryptone–yeast extract broth (UFTYE) for 3 days, at 10% CO_2_ and 37 °C, and exposed to 10% sucrose for 1 min, 8×/day. After incubation, the supernatant was removed and the wells were washed twice with PBS (200 μL/well) to remove planktonic and loosely bound cells. The attached bacterial cells were then stained with 0.1% crystal violet for 15 min and dissolved in 96% ethanol to remove the bound dye from the stained cells. The dye bound to biofilm was re-solubilized with 150 μL of 33% acetic acid and the optical density (OD) of each well was measured at 570 nm by a microplate reader (BioTek, Germany) [23]. The following controls were included in each MBIC test plate: 1) 0.2% chlorhexidine (CHX; positive control); 2) *S. mutans* in medium (negative control); 3) medium only (sterility control). MBIC was defined as the lowest concentration of an antimicrobial agent required to inhibit the formation of biofilms [21].

### Human saliva collection

Whole saliva samples were collected from a healthy volunteer after overnight fasting and pooled into sterile bottles. The collected saliva was then centrifuged at 8000 rpm for 15 min at 4 °C, sterilized using a syringe filter with a 0.22 μm pore size, and coated onto wells of the 96-well microtiter plates for biofilm formation assays [6].

### Biofilm formation and treatment procedure

The coated plates with saliva were incubated at 4 °C for 60 min and were washed twice with sterile phosphate-buffered saline (PBS) followed by air drying for 30 min. The overnight culture of *S. mutans* was then inoculated in a semi-defined biofilm medium (BM) [24] containing a final concentration of 18 mM glucose and 2 mM sucrose (BMGS) [25]. Bacterial suspensions (100 μL) were added to the wells and the microtiter plates were incubated for 72 h at 37 °C in the presence of 5% CO_2_ to form the mature biofilms. Finally, the formed biofilms in the experimental groups were treated with experimental groups as follow:

A. N-QCT: The 96-well microtiter plate containing preformed *S. mutans* biofilms was treated with 200 μL of N-QCT at sub-MBIC doses in the dark at room temperature for 5 min. The biofilm structures were washed twice with 200 μL of PBS to remove the planktonic bacterial cells.
B. Blue laser: The preformed *S. mutans* biofilms were exposed to a blue laser (Laser Diode, ASHA, Iran) at a wavelength of 405 ± 10 nm (according to the wavelength obtained from UV-Vis) and output intensity of 150 mW/cm^2^ for 60 s.

The polystyrene microplate has 0% and less than 10% light absorption and reflection, respectively, at wavelengths between 400 and 1000 nm [26, 27]. The absorbance value of polystyrene microplate wells containing distilled water is the same as the empty ones [28] which reveals the advantage of applying polystyrene microplate for absorbance measurements in the visible light region of the spectrum including 405 ± 10 nm which was used in the current study [28]. To prevent beam reflection from the table top and transmission of light to wells around the test wells during irradiation in blue laser and aPDT groups, sheets of black paper were used under the microplates and filled the neighboring test wells with methylene blue, respectively. The probe of the laser was fixed 2 mm above the top surface of the microplate by a stand [29].

III. aPDT: The preformed *S. mutans* biofilms were treated by sub-MBIC doses of N-QCT similar to group A and were then exposed with a blue laser similar to group B.
IV. Positive control: 200 μL of 0.2% chlorhexidine (CHX) was added to *S. mutans* biofilms and incubated at room temperature for 5 min.
V. Negative control: 200 μL of normal saline was added to *S. mutans* biofilms and incubated for 5 min at room temperature.

### Measurement of intracellular ROS generation

The generation of intracellular ROS following each treatment was evaluated using fluorescent2′,7′-dichlorofluorescein diacetate (DCFH-DA) method as follows [30]:

#### N-QCT

The 96-well microtiter plate containing preformed *S. mutans* biofilms was treated with 200 μL of N-QCT at sub-MBIC doses in the dark at room temperature for 5 min. The biofilm structures were washed twice with 200 μL of PBS to remove the planktonic bacterial cells. The biofilm cells were then incubated with 10 μM DCFH-DA (200 μL) for 1 h. The fluorescence intensity of DCF was then quantified with excitation and emission wavelengths of 485 and 530 nm, respectively.

#### Blue laser

ROS detections following irradiation were carried out in 1 × 1 × 3 cm glass cuvettes containing preformed *S. mutans* biofilms. The biofilm structures were washed twice with 2000 μL of PBS to remove the planktonic bacterial cells. After treatment of *S. mutans* biofilms with 10 μM DCFH-DA for 1 h. The blue laser probe stood on the top surface of the cuvette. This permitted irradiation during the acquisition of a fluorescence signal in real-time. The blue laser was switched on immediately before the beginning of fluorescence acquisition. The fluorescence intensity of DCF was then quantified with excitation and emission wavelengths of 485 and 530 nm, respectively.

#### aPDT

ROS detections following photodynamic reaction were carried out after treatment *S. mutans* biofilms with sub-MBIC doses of N-QCT similar to the N-QCT group and were then exposed with a blue laser similar to the blue laser group as mentioned above.

#### Positive and negative control groups

ROS assessment was performed in the positive control and negative control groups as in the N-QCT group, except that 0.2% CHX and sterile normal saline were used instead of N-QCT in the positive and negative control groups, respectively.

### Assessment of biofilm viable cells by colony count

Media was removed from all wells after treatment of *S. mutans* biofilms according to the experimental design described. The formed biofilms were washed twice with PBS (200 μL). Next, 100 μL of sterile normal saline solution was added to wells containing biofilm, and the biofilm cells were suspended by vigorous pipetting. The suspended biofilm was transferred to a new microtiter plate followed by 5-fold dilutions prepared in BHI broth. Immediately, 10 μL of each well-containing dilution series were cultured onto BHI agar and the plates were incubated at 37 °C for 24 h. Eventually, the log_10_ CFU/mL were determined as the previous study [31]. Bactericidal activity was defined as a reduction of at least ≥3 log_10_ (99.9%) of the bacterial count (CFU/mL) in the original inoculum [32]. The percentage of microbial cell reduction after each treatment was calculated using the following equation:

$$P=\left(1-10-L\right)\times 100$$

P: is the percent reduction.

L: is the log reduction.

### Assessment of biofilm biomass by colorimetric assay

After treatment of *S. mutans* biofilms based on the study groups, the microtiter plates were lightly washed twice with 200 μL/well of PBS to remove planktonic and loosely bound cells. The remaining cells were then stained with 0.1% crystal violet as described above, and the OD of the suspensions was measured spectrophotometrically at 570 nm.

### Assessment of metabolic activity of biofilm cells by flow cytometry

Mature *S. mutans* biofilms were prepared, treated as described above, and resuspended. After that, 100 μL of biofilm suspensions were transferred to the microtubes and mixed with 10 μL of Syto9 (20 μM) for 15 min, and later, 10 μL of 800 μM propidium iodide (PI) for 5 min. Then, samples were immediately processed and analyzed by flow cytometry (Becton Dickinson, USA). The sample was excited at 488 nm and the emission was registered using the FITC channel for Syto 9 (530/30) and PerCP channel (670/LP) for PI [33]. The biofilm cell viability was reported in percentage in relation with the control cells (untreated biofilm cells) [34].

### In silico analysis of CSP in *S. mutans*

Molecular modeling and in silico characterization of the structural subunit of CSP were evaluated. The amino acid sequences for the virtual search were selected using the CSP of sequence CAL29411.1. Alignment with a Protein Basic Local Alignment Search Tool-NCBI (BLAST-P) was conducted in Protein Data Bank (PDB) entries to retrieve and find similar sequences to CSP. Physicochemical properties of CSP were performed using UniProt. STRING (http://string-db.org) quantitatively assimilated the protein-protein interactions (PPI) network and ranked their significance or validity as targets.

### Docking analysis

The two-dimensional structure of QCT was downloaded from the PubChem database. The Surflex-Dock program was used to build the interaction model between QCT and CSP and SwissDock was used to study the binding orientation of QCT into the CSP structure. Best protein-ligands complexes were selected according to the scoring function of the SwissDock fitness score.

### Quantification of gene expression using reverse transcription quantitative real-time PCR (RT-qPCR)

Immediately after treatment, the cells were pelleted by centrifugation and total RNA extraction was performed using the super RNA extraction Kit (AnaCell, Iran). Total RNA (150 ng) was reverse transcribed in a 10 μL cDNA reaction volume RevertAid First Strand cDNA Synthesis Kit (Thermo Scientific GmbH, Germany) based on the manufacturer’s instructions following the deletion of residual genomic DNA using RNase-free DNase I treatment (Thermo Scientific GmbH, Germany). The gene-specific primers designed using Primer3Plus software version 4.0 (http://bioinfo.ut.ee/primer3/) are listed in Table 1. The normalization of RT-qPCR data was carried out using the *16S rRNA* gene as an internal control. Distilled water was used as a negative control in this study. RT-qPCR was conducted using Line-GeneK Real-Time PCR Detection System and Software (Bioer Technology, Hangzhou, China), with control and standard as described previously [35]. Fold differences in mRNA expression were determined using Livak and Schmittgen [36] method and down- and upregulation were considered significant when the relative expression was decreased or increased ≥ two-folds.

**Table 1:** Primer sequence for RT-qPCR analysis of various genes in *S. mutans*

Gene	Primer Sequence (5′–3′)	Product size (bp)^**a**^
***comA***		
Forward	TTGTTGGCGCAAAACAATAA	174
Reverse	AATGGTTTCCATCCCATTGA	
***comB***		
Forward	CCAGTCCAAACCGTCAGACT	164
Reverse	GCTGCTTTCCTTGTCTTTCG	
***comDE***		
Forward	ACAATTCCTTGAGTTCCATCCAAG	81
Reverse	TGGTCTGCTGCCTGTTGC	
***gtfB***		
Forward	TGTTGTTACTGCTAATGAAGAA	103
Reverse	GCTACTGATTGTCGTTACTG	
***16S rRNA***		
Forward	CCTACGGGAGGCAGCAGTAG	121
Reverse	CAACAGAGCTTTACGATCCGAAA	

^a^*bp* base pair

### Statistical analysis

All experiments were repeated at least three times. Data were analyzed by one-way ANOVA followed by Tukey post hoc test using Statistical Package for the Social Sciences (SPSS) software version 25.0. All data are expressed as means ± standard deviation (SD), and *P*-value < 0.05 was considered statistically significant.

## Results

### Confirmation of N-QCT synthesis

**Fig. 1:**
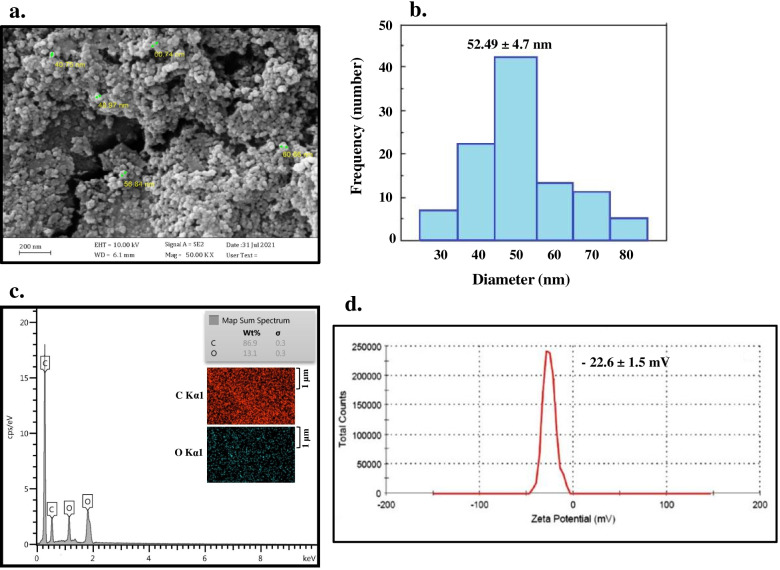
Characterization of synthesized nano-QCT (N-QCT): **a** Field emission scanning electron microscope (FESEM) image (Scale bar = 2 μm), **b** The size distribution profile of N-QCT, **c** Elemental mapping of N-QCT

N-QCT had a spherical morphology with an average diameter of 52.49 ± 4.7 nm (Fig. 1a). The hydrodynamic size of the N-QCT was within 30-80 nm as observed from dynamic light scattering (DLS) measurement (Fig. 1b). Appearances of characteristic Kα lines of C and O in the context of the sample confirmed the presence of N-QCT (Fig. 1c). The average surface charge of N-QCT was − 22.6 ± 1.5 mV (Fig. 1d). Additionally, the EE and DL capacity ranged from 74.12 to 88.25% and from 5.36 to 17.20%, respectively.

**Fig. 2:**
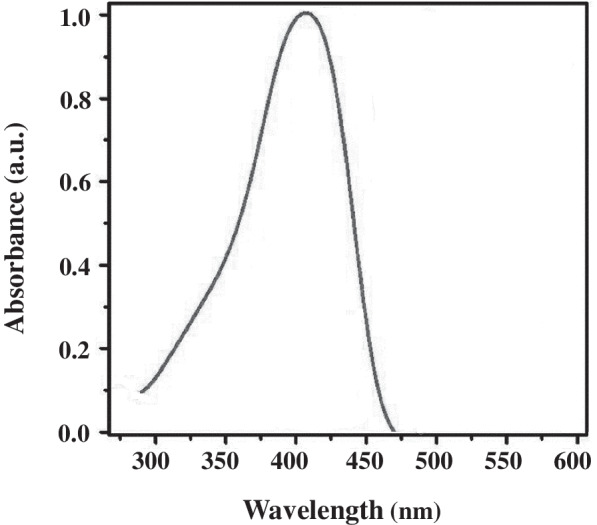
Ultraviolet–visible spectroscopy of N-QCT

The UV–vis spectrum of N-QCT is presented in Fig. 2. Accordingly, the absorption wavelength of N-QCT was found to be 405 nm.

### Effect of N-QCT on cell viability

**Fig. 3:**
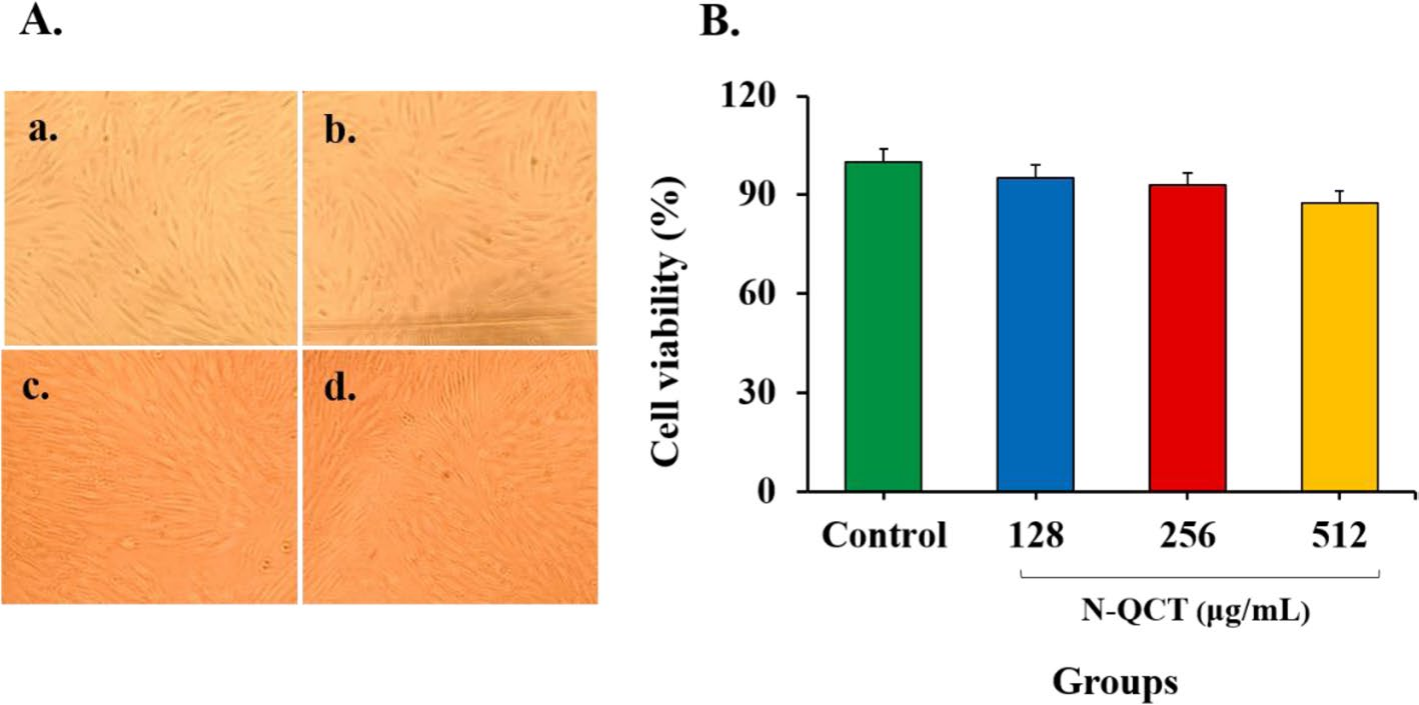
Effects N-QCT on cell viability of human gingival fibroblast cells: **A** Inverted light microscope images of treated cells with varying concentrations of N-QCT (magnification × 40); **a** Control (Untreated cells), **b** 128 μg/mL, **c** 256 μg/mL, and **d** 512 μg/mL, **B**. The mean percentage viability versus concentrations of N-QCT using MTT assay

The functional effect of N-QCT on HGF as normal fibroblast cells was investigated using the MTT assay. According to the data, treatment of the cells with 128, 256, and 512 μg/mL of N-QCT for 24 h had a non-considerable adverse effect on HGF cells (*P* > 0.05; Fig. 3).

### MBIC of N-QCT

**Fig. 4:**
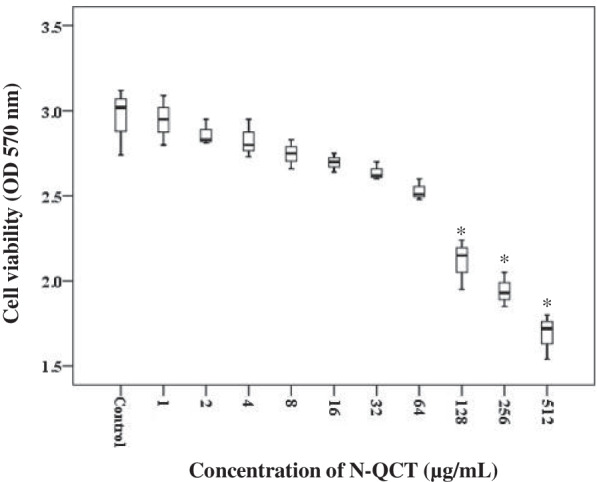
Anti-biofilm effect of N-QCT at the different concentrations against *S. mutans*. *Significantly different from the control group (no treatment), *P* < 0.05

N-QCT at 128-512 μg/mL concentrations statistically reduced *S. mutans* biofilm when compared to untreated biofilm cells (*P* < 0.05; Fig. 4), whereas there was no significant effect on the biofilm destruction when N-QCT was decreased from 64 to 1.0 μg/mL. According to the findings, at least a concentration of 128 μg/mL of N-QCT as MBIC was required to destroy the increasing growth of the biofilm.

### Generation of intracellular ROS

**Fig. 5:**
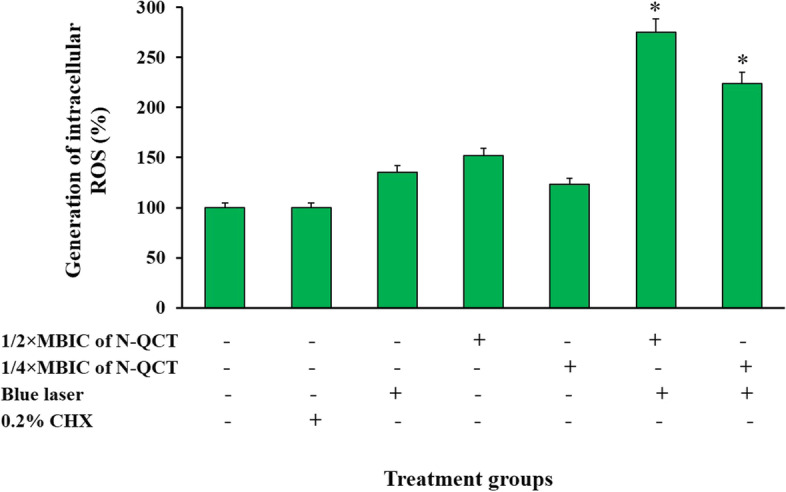
Effects of different treatment groups on the production of intracellular reactive oxygen species (ROS) in *S. mutans*. *Significantly different from the control group (no treatment), *P* < 0.05

N-QCT at 1/2× MBIC (64 μg/mL) and 1/4 × MBIC (32 μg/mL), as well as blue laser irradiation led to an increase in ROS levels compared to the non-treated cells (1.52-, 1.23-, and 1.35-folds, respectively). Maximum ROS production was observed in the aPDT groups (Fig. 5), with application of 1/2× and 1/4× MBIC of N-QCT leading to 2.75- and 2.24-fold higher ROS production, compared to control cells (untreated cells; *P* < 0.05).

### Anti-biofilm effects of treatment groups against *S. mutans* biofilms

**Fig. 6:**
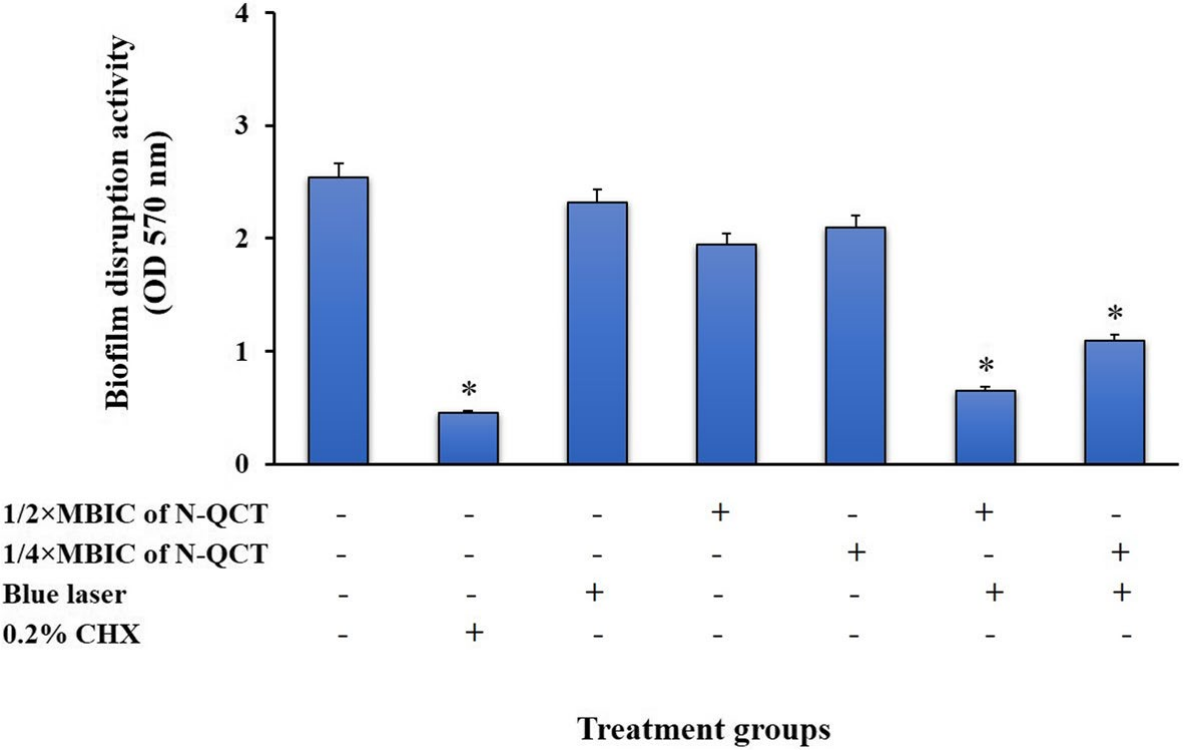
Biofilm disruption effects of different treatment groups against *S. mutans*. *Significantly different from the control group (no treatment), *P* < 0.05

Significant degradation of preformed biofilms was observed in the group treated with aPDT using sub-MBIC doses of N-QCT plus irradiation of blue laser for 60 s (*P* < 0.05; Fig. 6). Although the preformed biofilms treated with 1/2× and 1/4× MBIC of N-QCT and blue laser also showed similar results with decreased cell viability, the rate of biofilm degradation was not considerable (*P* > 0.05). Also, the results showed no significant differences between aPDT using 1/2 × MBIC of N-QCT and 0.2% CHX in biofilm inhibition (*P* > 0.05).

**Fig. 7:**
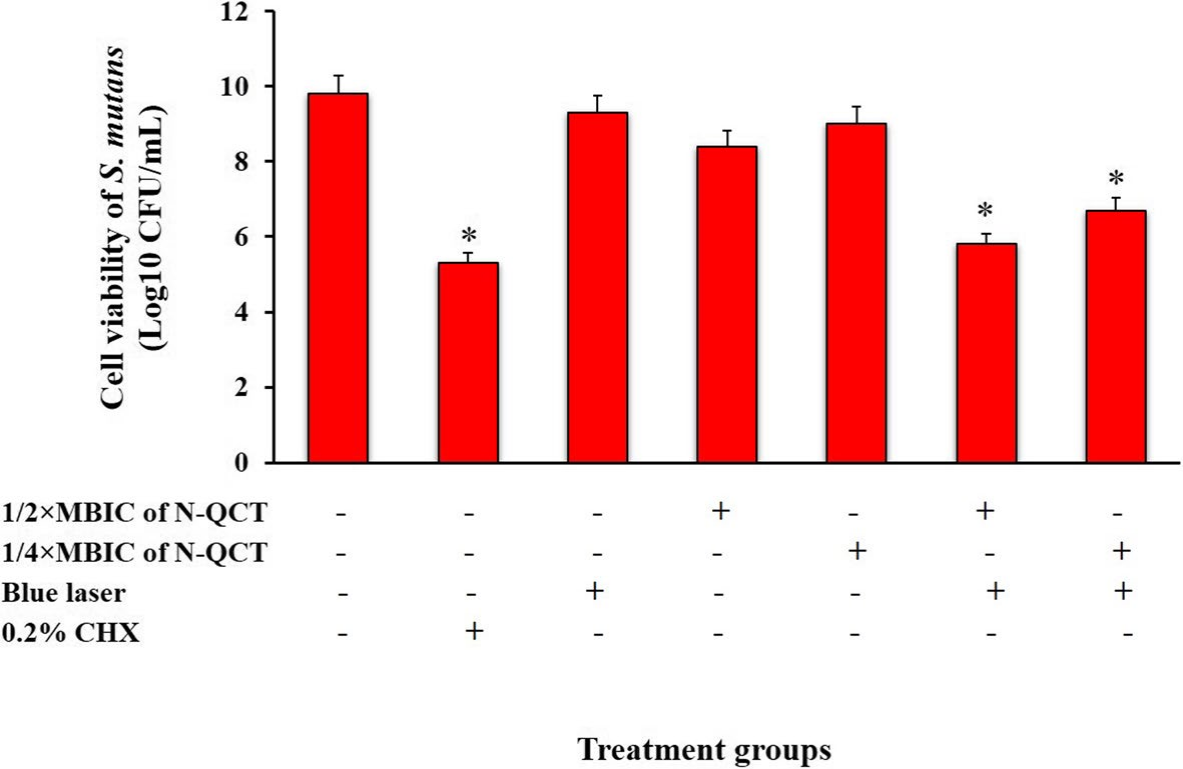
Effect of different treatment groups on cell viability of *S. mutans*. *Significantly different from the control group (no treatment), *P* < 0.05

The crystal violet staining of the biofilms also confirmed the log_10_ CFU/mL results. As shown in Fig. 7, a dense biofilm matrix was seen in the control group, while dead cells increased in the biofilm matrix treated with aPDT (both concentrations) and 0.2% CHX (*P* < 0.05).

Also, Log_10_ CFU/mL reduction and percent reduction following each treatment were shown in Table 2. Our results demonstrated that there was a significant reduction in cell viability of *S. mutans* following aPDT groups using 1/2× and 1/4 × MBIC of N-QCT, and 0.2% CHX (*P* > 0.05).

**Table 2:** Log_10_ CFU/mL, Log_10_ CFU/mL reduction, and percent reduction of treated *S. mutans* biofilm following each treatment

Groups	Log_**10**_ CFU/mL	Log_**10**_ CFU/mL reduction	Percent reduction (%)
**Control**	9.8	–	–
**0.2% CHX**	5.3	−4.5	99.99
**Blue laser**	9.3	−0.5	68.41
**1/2 × MBIC of N-QCT**	8.4	−1.4	96.11
**1/4 × MBIC of N-QCT**	9	−0.8	84.22
**aPDT using 1/2 × MBIC of N-QCT**	5.8	−4.0	99.99
**aPDT using 1/4 × MBIC of N-QCT**	6.7	−3.1	99.92

### Biofilm metabolic activity of *S. mutans* by flow cytometry

**Fig. 8:**
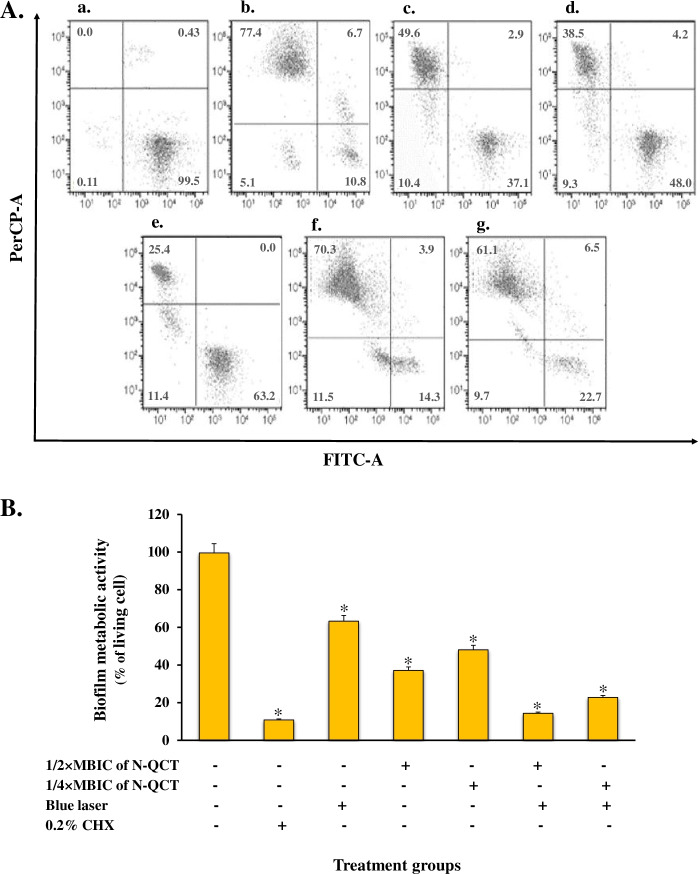
Effect of different treatment groups on biofilm metabolic activity in *S. mutans*; **A** measured by flow cytometry: **a** Untreated biofilm cells, **b** 0. 2% CHX, **c** N-QCT at 1/2 × MBIC, **d** N-QCT at 1/4 × MBIC, **e** Blue laser light, **f** aPDT using 1/2 × MBIC of N-QCT, and **g** aPDT using 1/4 × MBIC of N-QCT. **B** Percentage of biofilm living cells. *Significantly different from the control group (no treatment), *P* < 0.05

Flow cytometry was employed to measure metabolic activity in treated *S. mutans* biofilms. Figure 8A depicts the differentiation of live and dead cell populations as individual dot plots. All treatment groups significantly reduced the biofilm metabolic activity in *S. mutans* (*P* < 0.05). As demonstrated in Fig. 8B, both aPDT groups were more efficient in reducing *S. mutans* metabolic activity compared to the other treatment groups (Fig. 8B). The percentage of living cells was 14.3 ± 1.73% and 22.7 ± 2.45% in cells treated with laser plus 1/2× and 1/4 × MBICs of N-QCT, respectively. Blue laser irradiation also showed anti-metabolic activity compared to the control group but it was less than that observed in the N-QCT groups at 1/2 × MBIC (37.1 ± 2.06% live cells) and 1/4 × MBIC (48.0 ± 3.00% live cells) Fig. 8B).

### Molecular modeling and docking analysis

The sequence similarity and coverage results on CSP displayed that it is similar to the protein structure with accession number 2JHQ. The total score for protein alignment between CSP and 2JHQ was 62.4, with 95 and 28.89% query cover and identity, respectively. Basic information obtained from CSP at the NCBI GenBank database showed that it has 46 amino acids and its estimated structure weight and theoretical pI were 5211.06 Da and 9.70, respectively. The amino acid compositions of CSP are: Ala (A) 2.2%; Arg (R) 4.3%; Asn (N) 4.3%; Asp (D) 4.3%; Cys (C) 0.0%; Gln (Q) 2.2%; Glu (E) 6.5%; Gly (G) 8.7%; His (H) 0.0%; Ile (I) 8.7%; Leu (L) 13.0%; Lys (K) 13.0%; Met (M) 2.2%; Phe (F) 10.9%; Pro (P) 0.0%; Ser (S) 10.9%; Thr (T) 8.7%; Trp (W) 0.0%; Tyr (Y) 0.0%; and Val (V) 0.0%. The results obtained from UniProt revealed that CSP had 8 positively charged residues (Arg + Lys) and 5 negatively charged residues (Asp + Glu) with an aliphatic index of 86.96. Also, the instability index (II) and grand average of hydropathicity value were 20.99 and − 0.224, respectively. These results classify the protein as stable.

**Fig. 9:**
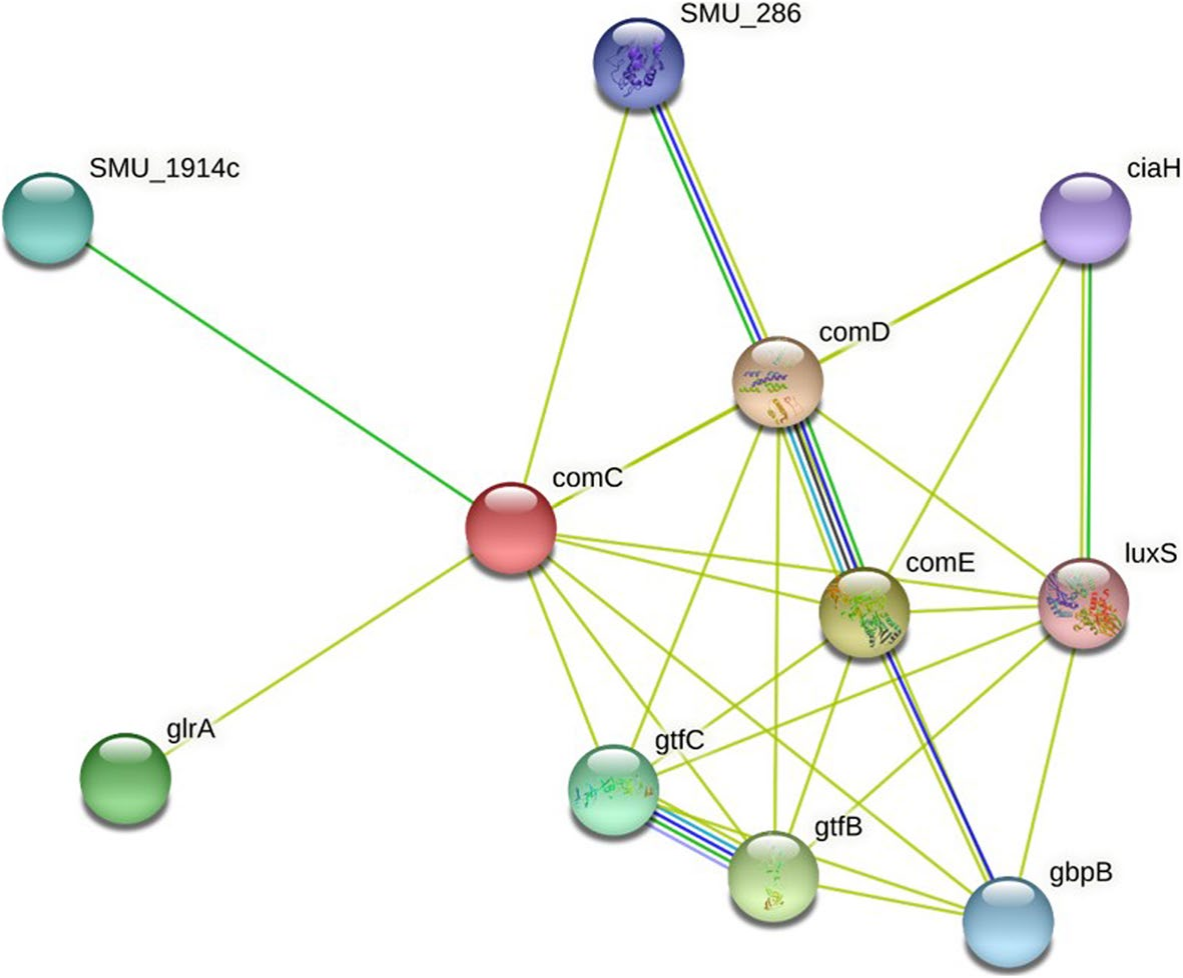
The PPI network of CSP protein identified in *S. mutans*

The PPI network of CSP was constructed. The main connected component including 10 nodes was formed. As shown in Fig. 9, each node is distinguishable from the other nodes based on degree value. Whenever the protein score is closer to 1.0 is an indication that this protein is near to CSP. The properties of each node based on analysis of the PPI network are tabulated in Table 3.

**Table 3:** The list of the properties of key nodes based on the PPI network analysis

Nodes	Predicted functional partners	Neighborhood	Gene Fusion	Co-occurrence	Co-expression	Experiments	Databases	Textmining	Score
**comD**	Two-component system, lyttr family, sensor histidine kinase agrc; Putative histidine kinase of the competence regulon							*	0.750
**comE**	Putative response regulator of the competence regulon, come; Response regulator of the competence regulon ComE							*	0.750
**gtfB**	Glucosyltransferase-i; Production of extracellular glucans, that are thought to play a key role in the development of the dental plaque because of their ability to adhere to smooth surfaces and mediate the aggregation of bacterial cells and food debris							*	0.711
**glrA**	ABC-2 type transport system ATP-binding protein							*	0.585
**gtfC**	Glucosyltransferase-si; Production of extracellular glucans, that are thought to play a key role in the development of the dental plaque because of their ability to adhere to smooth surfaces and mediate the aggregation of bacterial cells and food debris							*	0.516
**SMU_1914c**	Hypothetical protein; Uncharacterized protein	*							0.442
**gbpB**	Peptidoglycan dl-endopeptidase cwlo; Putative secreted antigen GbpB/SagA putative peptidoglycan hydrolase							*	0.429
**SMU_286**	Atp-binding cassette, subfamily c, bacteriocin exporter							*	0.429
**ciaH**	Two-component system, ompr family, sensor histidine kinase ciah; Putative histidine kinase sensor CiaH							*	0.428
**luxS**	Putative autoinducer-2 production protein luxs; Involved in the synthesis of autoinducer 2 (AI-2) which is secreted by bacteria and is used to communicate both the cell density and the metabolic potential of the environment. The regulation of gene expression in response to changes in cell density is called quorum sensing. Catalyzes the transformation of S-ribosylhomocysteine (RHC) to homocysteine (HC) and 4,5-dihydroxy-2,3-pentadione (DPD)							*	0.428

**Fig. 10:**
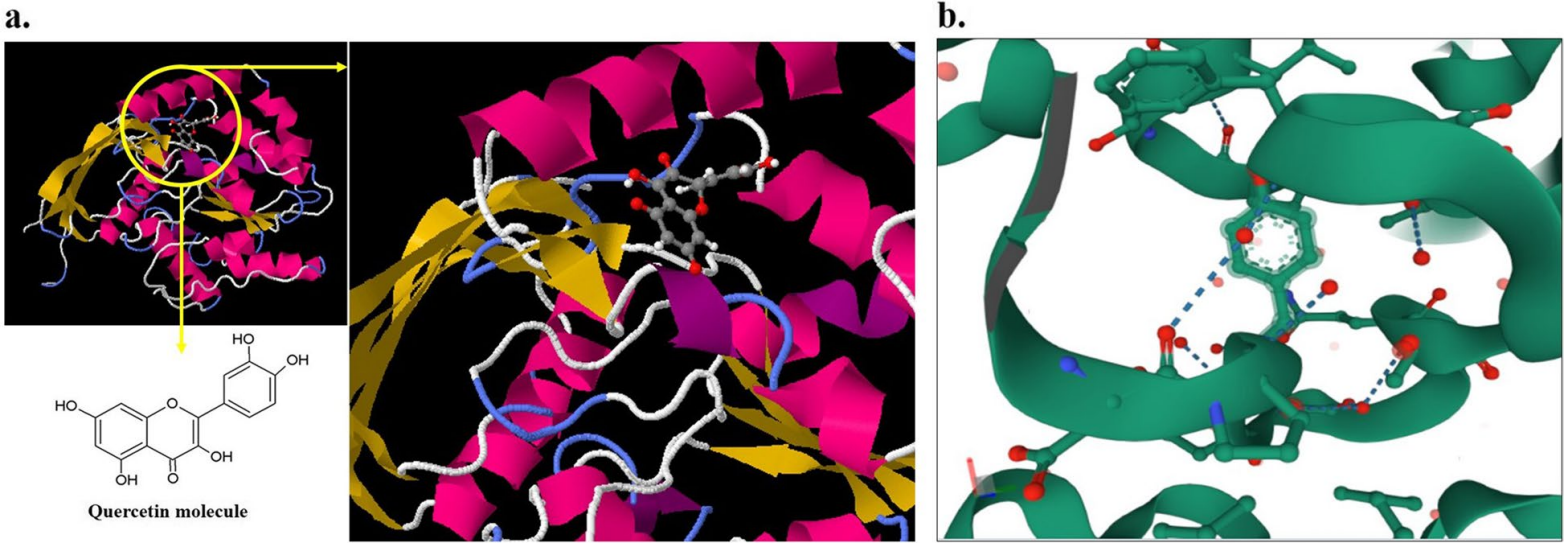
Molecular modeling of QCT-bound CSP. The residues of CSP and QCT are represented using a ball-and-tinctorial stick physical model: **a** Binding site of QCT in CSP, **b** Conformation of QCT in the binding site of CSP. The bonds between the ligand and the protein are presented by blue dashed lines

The molecular docking analysis was performed using SwissDock. The lowest binding free energy docked conformation of the best cluster was selected and analyzed. The best docking results are given in Fig. 10. As shown in Fig. 8a, the QCT molecule was located in the site formed by polypeptide helices. Figure 8b shows that the QCT molecule was adjacent to some residues in the hydrophobic cavity; oxygen atoms 7 and 10 in QCT formed hydrogen bonds with two hydrogen atoms in Tyr66 and Gly109. In addition, hydrogen atoms 6, 9, and14 formed hydrogen bonds with Ala 78, Arg177, and His 202, respectively.

### Gene expression profiling using RT-qPCR

**Fig. 11:**
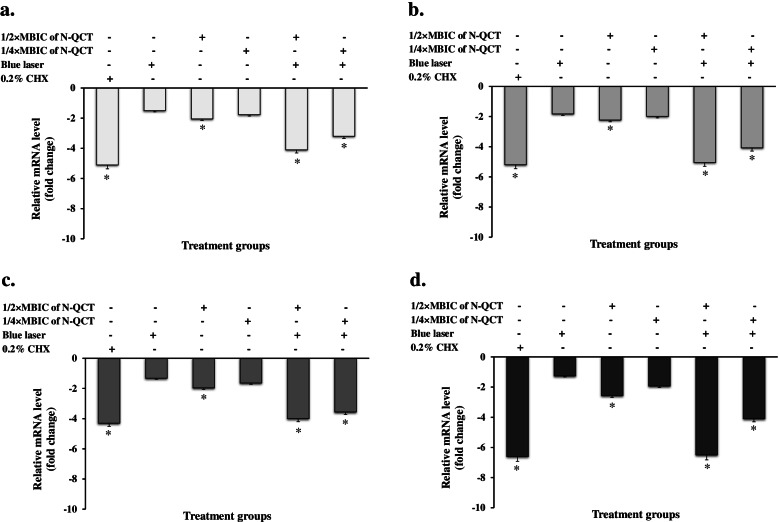
Effect of different treatment groups on the expression of various genes involved in quorum sensing of *S. mutans*: **a** *comA*, **b** *comB,* **c** *comDE,* and **d** *gtfB*. *Significantly different from the control group (no treatment), *P* < 0.05

Gene expression analysis using RT-qPCR in *S. mutans* revealed that the aPDT groups downregulated the QS signals-related genes. As shown in Fig. 11, 1/2 × MBIC of N-QCT plus blue laser could decrease the gene expression levels of *comA, comB, comDE,* and *gtfB* by 4.1-, 5.05-, 4.0-, and 6.5-fold, respectively (*P* < 0.05). Also, a 3.2-, 4.08-, 3.56-, and 4.1-fold reduction in the expression of *comA*, *comB*, *comDE*, and *gtfB* genes was observed in the biofilm cells treated with aPDT using 1/4× MBIC of N-QCT-treated (P < 0.05). In contrast, 1/2 × MBIC of N-QCT, 1/4 × MBIC of N-QCT, and blue laser alone did not show any significant effect on the expression of QS genes of *S. mutans* biofilm. (*P* > 0.05). It is noteworthy that there is no significant difference in the reduction of genes expression between aPDT and 0.2% CHX groups (*P* > 0.05).

## Discussion

Many microorganisms in dental plaque biofilm deposited on the tooth surface have been found to be associated with dental caries [37]. Extensive investigations showed that *S. mutans* was considered as a primary etiologic agent responsible for dental caries due to its ability to form biofilm and to provide adhesion of bacteria to each other and to the tooth enamel [38, 39]. So, the inhibition of *S. mutans* biofilm formation or degradation/dispersal of a formed biofilm could be the main target to forestall dental caries. Using hand or rotary instruments not only leads to incomplete removal of the caries and insufficient elimination of residual microorganisms but can also cause damage to the pulp, resulting in pain and weakening of the tooth structure [40]. On the other hand, chemical antimicrobial agents can cause various side effects and lead to the emergence of antimicrobial resistance due to overdose and prolonged administration. Therefore, in recent years, plant-derived natural products, as anti-biofilm agents, have been considered to be an appealing option for overcoming virulence biofilm characters [41].

According to the literature, QCT with broad-spectrum antimicrobial effects has been also used to prevent and treat various infectious bacterial diseases. Qin et al. [42] found that QCT inhibited *Staphylococcus aureus*, *Escherichia coli*, and *Pseudomonas aeruginosa* with minimum inhibitory concentrations (MICs) of 0.0061, 0.0242, and 0.0121 μmol/mL, respectively. In another study, Wang et al. [14] evaluated the bacteriostatic effect of QCT as an antibiotic alternative on four kinds of bacteria in vitro. They reported QCT had significant antibacterial activity against *E. coli*, *Salmonella enterica* Typhimurium, *S. aureus*, and *P. aeruginosa* with MICs of 0.0082, 0.0072, 0.0068, and 0.0085 μmol/mL, respectively. Nevertheless, QCT generally exhibits low bioavailability due to its poor aqueous solubility. To address this problem, various promising approaches, such as pharmaceutical nanotechnology have been developed that can effectively improve the bioavailability of QCT by enhancing its solubility, dissolution rate and permeability, and/or delivering them directly to their physiological targets [43]. In the present study, we used 1% of DMSO for the preparation of the N-QCT solution. However, in vitro studies have shown a lower solubility of quercetin. Kakran et al. [44] have shown the saturation solubility of coarse QCT was extremely low being only 2.84 ± 0.03 μg/mL while forming nanostructure enhanced the saturation solubility of QCT approximately nine times to 25.59 ± 1.11 μg/mL. The saturation solubility of the QCT-nanostructure is persistent and remains almost the same for seven days [44]. Usually, the existence of micrometer-sized crystals (acting as nuclei for crystallization) causes the saturation solubility to decrease by re-crystallization (growth of microcrystals being present). In the N-QCT structures, the microcrystals are present in a limited amount or absent, and hence, the saturation solubility remains unchanged. This is favorable for oral administration [44].

In this study, after confirmation of the synthesized N-QCT properties, its cytotoxic effect was investigated. We used MTT assay to determine the potential toxicity of N-QCT on the HGF cell line and found no cytotoxic effect. In addition, the biofilm inhibitory concentration of N-QCT against *S. mutans* biofilm was evaluated and according to the results, both minimum (128 μg/mL) and maximum (512 μg/mL) biofilm inhibitory concentrations, decreased the biofilms of *S. mutans* by 28.7 and 43.2%, respectively.

Several investigations evaluated the anti-cancer effects of QCT by reducing the cell viability of different cancer cell lines during aPDT [45–49]. To the best of our knowledge, this is the first study that has determined the effects of N-QCT-mediated aPDT on *S. mutans* biofilm. We calculated the MBIC of N-QCT (128 μg/mL) and assessed the effectiveness of its 1/2 and 1/4 values alone and in combination with blue laser on *S. mutans* biofilm. It was found that aPDT using 1/2× and 1/4 × MBIC of N-QCT disrupted the biofilm by 74.40 and 57.08%, respectively probably due to intracellular ROS generation.

In microbial cells ROS are produced by oxidative phosphorylation involves the transport of protons across the inner cell membrane by means of the electron-transport chain that occurs during the processes of respiration in normal metabolisms and under exogenous stress conditions. ROS has a dual role; whether they will act as signaling or harmful factors depends on the balance between ROS production and disposal at the right place and time. ROS are intrinsic to cellular functioning including cell homeostasis and signaling and are present at stationary and low levels in microbial cells. On the other hand, ROS as a highly reactive chemical agent can cause irreversible damage to DNA, oxidize and modify some cellular components, and prevent their original functions. Oxygen toxicity can arise both from the inefficient elimination of ROS by the antioxidant system and uncontrolled production [50]. Our results suggest that ROS over natural production in *S. mutans* cells following N-QCT-based aPDT reduces metabolic activity, preformed biofilms, and relative expression levels of the virulence genes, which is a feasible avenue to reducing *S. mutans* population as a main cariogenic bacterium.

Although the biofilms cells treated with sub-MBIC doses of N-QCT and blue laser alone showed decreased cell viability, the rate of biofilm disruption was not significant. In addition, we investigated the biofilm metabolic activity of treated *S. mutans* bacteria using flow cytometry. All treatment groups significantly reduced the biofilm metabolic activity of *S. mutans*; however, the reduction was more prominent in the aPDT groups. These results are consistent with those of previous studies in which aPDT was reported to exhibit an inhibitory effect on the metabolic activity of *S. mutans*. We showed that N-QCT had the ability to produce ROS, which probably led to the significant inhibition of the microbial metabolic activity.

The current study showed a discrepancy between metabolic activity and log_10_ CFU/mL as a tool for assessing cell proliferation so that a 4 log (40%) reduction was observed in 1/2 × MBIC of N-QCT treated with the light group, whereas only 80% were metabolically inactive in flow cytometry. The results of the current study are consistent with a report in which daunorubicin led to cell detachment but not to fragmentation of cells and they continued to convert MTT to formazan resulting in an apparent overestimation of cell viability when applying MTT. However, MTT was originally developed to measure chemical reagent cytotoxicity, but the literature review shows that mainly metabolic assays including MTT were used to quantify proliferative activities but such assays may not accurately reflect proliferation due to non-linear and miscorrelating changes in metabolic activity and cell number over time in culture. The obvious limitation of metabolic assays is that cellular metabolic activity varies greatly throughout the growth cycle of cells [51]. Tetrazolium salt-based assays including MTT do not measure the cell number of viable cells in a culture or their growth, but more a set of enzyme activities, that are related in various ways to the cellular metabolism.

In a major MTT-based cytotoxicity study [52], daunorubicin led to cell detachment but not to fragmentation of cells; however, the treated cells continued to reduce MTT to formazan resulting in an overestimation of cell viability. This disadvantage is common in vitro cell-based assays, warranting precise interpretation of the results. It could therefore be useful to monitor the cell counting including colony counting (CFU/ml) or live/dead staining procedure as a visual confirmation of cell viability in addition to the metabolic assay to validate the results.

On the other hand, generally metabolic activity is higher in actively proliferating microbial cells under optimal growth conditions when compared with treated cells. Reduction in microbial cell metabolic activity is the use of a defense mechanism to withstand sub-lethal external stress doses; surviving cells grow in the presence of the sub-lethal stress doses. This phenotype of the microbial cells is not inherited, and cells revert to the wild-type phenotype once the stress is removed. Microbial cells with reduced metabolic activity produce fewer targets of stress and can continue their viability. After an initial die-off of the microbial population following treatment, surviving cells (are known as persister cells) with a slowly increasing population density due to their reduction of metabolic activity [53]. Furthermore, persister cells are a subpopulation of bacteria within a larger antimicrobial agents-susceptible population that display decreased susceptibility to microbicidal agents through mechanisms involving metabolic repression [54]. Kwan et al. [55] have confirmed this evidence by demonstrating that persister cells reduce proton motive force, transcription, and translation, as well as Shah et al. [56], revealed reduced metabolic activity in persister cells using green fluorescent protein as a reporter under the control of a ribosomal promoter.

One of the most important advantages of successful aPDT targeting is the localization of the PS in the target site. For this purpose, molecular modeling and docking analysis were performed to confirm the connection of QCT to CSP. In silico findings showed that CSP is a stable protein with both positively and negatively charged residues. On the other hand, we found that the QCT molecule was located in the site formed by polypeptide helices of CSP, and oxygen and hydrogen atoms formed hydrogen bonds with CSP residues.

As previously proven, the biofilm formation in *S. mutans* is regulated by a QS signaling system that moderates the expression of virulence factors in a cell density-dependent manner [2, 57–59]. Therefore, inhibition of this system results in the reduction of *S. mutans* biofilm development and can consequently decrease the cariogenic process. Hence, disruption of *S. mutans* QS system has been proposed as a new anti-biofilm infection approach. Using QQ is considered to be one of the most promising methods for disrupting the QS systems and attenuating bacterial virulence [60, 61]. As Mion et al. [60] reported, QQ not only constitutes an interesting therapeutic strategy to fight against bacterial infections but also restricts the consequences of antibiotic resistance. It has been suggested that aPDT can be applied as a useful QQ strategy in enhancing the microbicide effects and in reducing drug resistance [16–18, 62, 63].

Our study examined the effect of QQ of *S. mutans* via aPDT on various QS-regulated genes. The QS pathway in *S. mutans* encompasses several genetic loci such as *comCDE* and *comAB*. The genes evaluated in this study are reported to be involved in the QS of *S. mutans* that further affect major virulence factors like biofilm formation. We found that the groups treated with blue laser plus 1/2× and 1/4 × MBIC of N-QCT, significantly downregulated the QS signal-related genes (*comA*, *comB*, and *comDE*). There was also a reduction in *gtfB* expression which is a gene involved in biofilm formation of *S. mutans*. The findings of the current investigation suggest that aPDT with a minimum concentration of N-QCT along with blue laser irradiation has the ability to perform QQ and through generation of large amounts of intracellular ROS is able to disrupt the microbial biofilm, reduce the metabolic activity of *S. mutans*, and downregulate the expression of genes associated with the QS signal system. Further studies are required to achieve a better understanding of QQ in microbe-microbe and host-pathogen interactions.

In RT-qPCR, CHX was used as a treatment (positive) control. The current study demonstrated that there is no significant difference in the reduction of genes expression between N-QCT mediated aPDT and CHX groups, which indicates N-QCT mediated aPDT is as effective as CHX in reducing virulence genes expression without CHX́ s side effects. CHX was introduced in dentistry in 1954 as a broad-spectrum microbicide effective against Gram-negative and Gram-positive bacteria [64]. However, it requires a high concentration of 0.2% to achieve effective results. Application of CHX has been limited due to many of its side effects including teeth staining, calculus buildup, and metallic aftertaste. Therefore, an antimicrobial strategy that robustly reduces or eliminates the microbial biofilms, while maintaining their biocompatibility, is highly desirable. The results of the current study showed that the effect of N-QCT mediated aPDT is as effective as the CHX in reducing genes expression and considering that aPDT is more bio-compatible, it can be considered an adjunctive treatment. We acknowledge that further evaluation of the antimicrobial activity of N-QCT mediated aPDT against additional common cariogenic bacteria, such as lactobacillus spp., will strengthen our study findings.

## Conclusion

Collectively, the data of the present study show that the combination of blue laser and N-QCT at low concentrations, target genes involved in the QS pathway of *S. mutans* making it a favorable QQ strategy. N-QCT-based aPDT may provide a potential adjuvant treatment to the currently used anti-biofilm approaches to prevent the incidence of dental caries.

## Data Availability

All data of this study are included in the manuscript. All figures are original images and have been used for the first time in this study. The primary datasets used during the current study in the section of in silico analysis about competence stimulating peptide (CSP) and quercetin (QCT) are available at https://www.ncbi.nlm.nih.gov/protein/115312774 and https://pubchem.ncbi.nlm.nih.gov/compound/5280459, respectively.

## Abbreviations

aPDT: Antimicrobial photodynamic therapy
CFU: Colony forming unit
CSP: Competence-stimulating peptide
MBIC: Minimum biofilm inhibitory concentration
N-QCT: Nano-quercetin
PS: Photosensitizer
QCT: Quercetin
QQ: Quorum quenching
QS: Quorum sensing
